# Treatment Options in Managing Infections Following Calcaneal Fractures: A Systematic Review

**DOI:** 10.3390/life16030528

**Published:** 2026-03-23

**Authors:** Giacomo Capece, Chiara Comisi, Guido Bocchino, Rocco Maria Comodo, Virginia Cinelli, Federico Moretti, Tommaso Greco, Giulio Maccauro, Carlo Perisano

**Affiliations:** 1 Orthopaedics and Trauma Surgery Unit, Catholic University of the Sacred Heart, 00168 Rome, Italy; giacomocapece97@gmail.com (G.C.); guido.bocchino@gmail.com (G.B.); roccocomodo96@gmail.com (R.M.C.);; 2 Orthopaedics and Trauma Surgery Unit, Department of Ageing, Neurosciences, Head-Neck and Orthopaedics Sciences, Fondazione Policlinico Universitario Agostino Gemelli IRCCS, 00168 Rome, Italy; 3 Orthopaedics and Trauma Surgery Unit, Pellegrini Hospital, 80134 Naples, Italy; 4 Department of Life Sciences, Health, and Healthcare Professions, Link Campus University, 00165 Rome, Italy

**Keywords:** calcaneal fracture, surgical site infection, fracture-related infection, ORIF, minimally invasive surgery, soft tissue complications, post-operative infection, foot and ankle trauma, systematic review

## Abstract

**Background:** Calcaneal fractures are complex injuries frequently associated with significant soft tissue damage and a high risk of post-operative complications, particularly infection. Despite advances in surgical techniques, infectious complications remain a major cause of morbidity and can severely compromise functional outcomes. The aim of this systematic review was to analyze the incidence, management strategies, and clinical impact of infectious complications following surgical treatment of calcaneal fractures. **Methods:** A systematic literature search was conducted in MEDLINE, Scopus, and Web of Science in accordance with PRISMA guidelines, including studies published up to May 2025. Randomized controlled trials and prospective and retrospective cohort studies involving adult patients surgically treated for calcaneal fractures and reporting post-operative infectious outcomes were included. Data extraction focused on patient demographics, fracture characteristics, surgical techniques, infection rates, microbiological findings, management strategies, complications, and functional outcomes. Methodological quality and risk of bias were assessed using the MINORS score. Due to substantial heterogeneity, results were synthesized descriptively. **Results:** Forty studies met the inclusion criteria, encompassing 5343 patients and 4638 surgically treated calcaneal fractures. Displaced intra-articular fractures predominated, with Sanders type II and III accounting for 79.8% of classified fractures, while Sanders type IV fractures represented 20.2% and were associated with higher complication rates. The overall post-operative infection rate was 9.4%, including 6.3% superficial surgical site infections and 3.0% deep infections. Open fractures accounted for 7.5% of reported cases and demonstrated markedly higher infection rates than closed injuries. Deep infections frequently required implant removal (62%), prolonged intravenous antibiotic therapy (100%), and additional surgical procedures (71%). *Staphylococcus aureus*, including methicillin-resistant strains, was the most commonly isolated pathogen. Functional outcomes were consistently worse in patients who developed infections. **Conclusions:** Infectious complications remain a clinically significant problem following surgical treatment of calcaneal fractures, particularly in severe fracture patterns, open injuries, and patients with relevant comorbidities. Deep infections are associated with substantial morbidity and inferior functional outcomes. Optimization of patient-related risk factors, careful surgical planning, and the selective use of minimally invasive approaches may help reduce infection risk. Further high-quality prospective studies with standardized outcome measures are needed to define optimal management strategies.

## 1. Introduction

Calcaneal fractures, involving the heel bone, pose a significant orthopedic challenge due to the complex anatomy of the calcaneus and its critical role in weight-bearing and foot stability [1]. These injuries often result from high-energy trauma, such as falls from heights or motor vehicle accidents, and can severely affect patients’ quality of life. Although calcaneal fractures are relatively rare, accounting for 1–2% of all skeletal fractures with an annual incidence of about 11.5 per 100,000 people, they can lead to substantial functional impairment if not properly treated [2,3,4].

The calcaneus, the largest bone in the foot, consists of a cancellous inner structure surrounded by a thin cortical shell [5,6]. These fractures typically occur due to traumatic compression forces that cause the collapse of the spongy bone [7,8]. Its anatomy makes it prone to compression fractures under axial loads, with intra-articular involvement happening in 70–75% of cases. Among hindfoot injuries, calcaneal fractures are the most common, representing 61% of tarsal fractures and about 2.6% of all fractures [9,10,11,12]. They often occur alongside other injuries, including ipsilateral talus or fibula fractures, lateral ligament complex injuries, fibular tendon dislocations, and flexor hallucis longus entrapment. Advanced imaging techniques, like MRI, can help in thoroughly assessing both bone and soft tissue damage [13,14].

Treatment options vary and include conservative approaches, closed or open reduction with internal or external fixation, arthroscopic-assisted techniques (ARIF), subtalar arthrodesis, and the use of bone grafts or substitutes [15,16]. Despite improvements in surgical methods, the best management approach remains debated and is usually customized based on fracture type, soft tissue condition, and individual patient factors. The risk of complications is notably high in open fractures, where traumatic wounds increase the chance of infection, osteomyelitis, or even amputation. Postoperative wound infections in closed fractures occur at rates around 5% for deep infections and up to 15% for superficial infections, especially with the extended lateral approach [17,18]. Due to the high risk of complications, particularly postoperative or post-traumatic infections, managing these patients is a major challenge. Infections can develop into chronic osteomyelitis, hinder fracture healing, and necessitate multiple surgeries, including partial or total calcanectomy or, in severe cases, amputation [19,20]. Various treatments have been suggested, such as aggressive surgical cleaning combined with local or systemic antibiotics, negative pressure wound therapy, antibiotic carriers, and reconstructive techniques for soft tissue coverage. However, the effectiveness, optimal timing, and long-term functional outcomes of these approaches are not well established.

A thorough review of current evidence is crucial to inform clinical decisions, improve infection control, and enhance limb salvage and functional recovery. This study aims to systematically analyze existing literature on infection management strategies after calcaneal fractures, focusing on clinical outcomes, complications, and functional results.

## 2. Materials and Methods

### 2.1. Search Strategy and Eligibility Criteria

**Figure 1:**
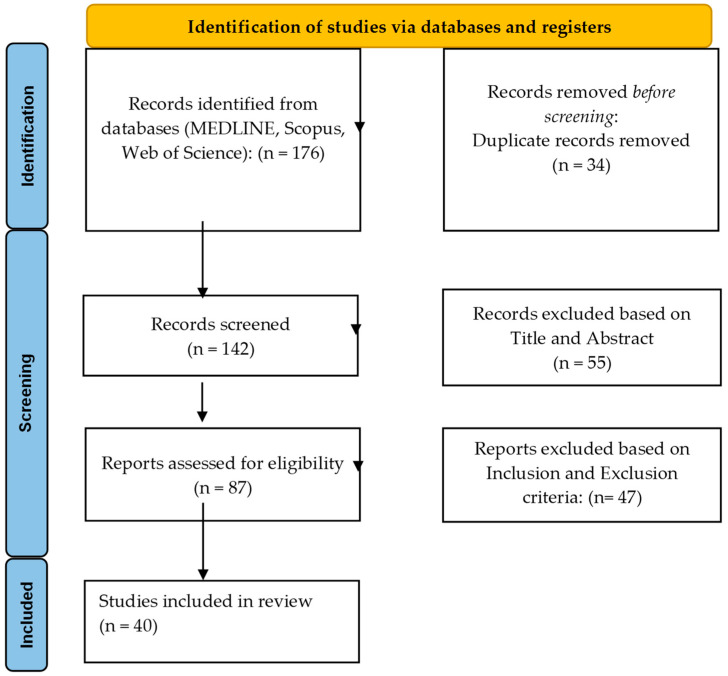
PRISMA flowchart.

This systematic review was conducted in accordance with the Preferred Reporting Items for Systematic Reviews and Meta-Analyses (PRISMA) guidelines [21] and included studies published up to May 2025 (Figure 1). The objective was to evaluate infectious complications, their management, and associated clinical outcomes in adult patients undergoing surgical treatment for calcaneal fractures.

A comprehensive literature search was performed in three electronic databases (MEDLINE, Scopus, and Web of Science). The search strategy combined terms related to calcaneal fractures and infection, and was developed using the following keywords: (“calcaneal fracture” [Title/Abstract] OR “calcaneus fracture” [Title/Abstract]) AND (“infection” [Title/Abstract] OR “infected” [Title/Abstract] OR “septic” [Title/Abstract] OR “wound complication” [Title/Abstract]) AND (“management” [Title/Abstract] OR “treatment” [Title/Abstract] OR “therapy” [Title/Abstract]). No language or time restrictions were applied.

To avoid overlap with other ongoing review studies, the protocol was registered in the International Prospective Register of Systematic Reviews (PROSPERO) prior to study submission (registration ID: 1287798).

Post-operative infections were defined according to the Centers for Disease Control and Prevention (CDC) criteria for Surgical Site Infections (SSIs), including superficial incisional, deep incisional, and organ/space infections [22]. When reported by the original authors, fracture-related infections were additionally considered consistent with the Fracture-Related Infection (FRI) consensus definition proposed by Metsemakers et al. [23,24]. Only studies explicitly reporting infections diagnosed on clinical, microbiological, and/or radiological criteria compatible with these definitions were included.

Studies were eligible for inclusion if they involved adult patients (≥18 years) with surgically treated calcaneal fractures who developed post-operative infection and reported a minimum follow-up of at least 12 months. Eligible designs included randomized controlled trials, prospective cohort studies, and retrospective cohort studies. Exclusion criteria comprised case reports, expert opinions, letters to the editor, previous systematic reviews or meta-analyses, studies investigating non-surgical management, studies with incomplete data or without quantitative infection outcomes, and studies lacking a clear infection definition. Only full-text articles were considered. Journal titles, authors’ names, and institutional affiliations were not masked during the selection process. When necessary, corresponding authors were contacted to clarify uncertainties or obtain missing information. A detailed summary of eligibility criteria is reported in Table 1.

### 2.2. Study Assessment and Data Extraction

Titles and abstracts of all retrieved records were independently screened by two reviewers (C.C. and G.C.). Full-text articles were obtained for all studies deemed potentially eligible or when eligibility was uncertain. Full-text eligibility was subsequently assessed by two independent reviewers (G.B. and R.M.C.) according to the predefined inclusion and exclusion criteria. Disagreements were resolved through discussion with a third author (T.G.). In addition, the reference lists of included articles were manually screened to identify further relevant studies not captured by the electronic search.

The risk of bias and methodological quality of included non-randomized studies were assessed using the Methodological Index for Non-Randomized Studies (MINORS) tool [25]. This instrument evaluates methodological domains related to selection processes, outcome assessment, follow-up adequacy, and reporting. Two authors (F.M. and C.C.) independently assigned MINORS scores, and disagreements were resolved by consensus. MINORS assessment was performed to characterize study quality and potential risk of bias and was not used as a criterion for study exclusion.

Data were systematically extracted from each study, including study design, sample size, patient demographics, fracture characteristics, details of the index surgical procedure, perioperative variables, infection rates and classification (superficial vs. deep when available), microbiological findings, management strategies for infectious complications, additional surgical procedures, complications, and functional outcomes. A third author (C.P.) independently verified extracted data to ensure accuracy and consistency.

### 2.3. Statistical Analysis

Descriptive statistics were used to summarize patient characteristics, fracture patterns, surgical techniques, and clinical outcomes. Continuous variables were reported as means and ranges when available, whereas categorical variables were expressed as absolute numbers and percentages using the appropriate denominator for each variable.

Statistical analysis was performed using IBM SPSS Statistics version 31.0.1.0 (IBM Corp., Armonk, NY, USA). Due to substantial clinical and methodological heterogeneity among the included studies, particularly regarding study design, outcome definitions, and follow-up duration, a formal quantitative meta-analysis was not feasible. Consequently, results were synthesized using a descriptive narrative approach.

## 3. Results

The systematic search identified 176 records across MEDLINE, Scopus, and Web of Science. After removal of duplicates, 142 studies were screened by title and abstract. Following full-text evaluation, 40 studies met the predefined inclusion criteria and were included in the final qualitative synthesis (Figure 1).

The included studies were published between 2001 and 2025 and consisted predominantly of retrospective observational cohort studies, accounting for approximately 80% of the sample, while the remainder included prospective cohort studies and randomized controlled trials. Overall, the studies analyzed 5343 patients with a total of 4638 surgically treated calcaneal fractures. Most studies originated from Asia and Europe, with China, the United Kingdom, and Italy being the most frequently represented countries. Mean follow-up duration ranged from 4 months to 10 years, with the majority of studies reporting a minimum follow-up of at least 12 months (Table 2).

### 3.1. Patient and Fracture Characteristics

The Sanders classification was reported in 31 of the 40 included studies, accounting for 3912 classified calcaneal fractures. Displaced intra-articular fractures constituted the vast majority of cases. Among classified fractures, Sanders type II fractures accounted for 1604 cases (41.0%), Sanders type III for 1517 cases (38.8%), and Sanders type IV for 791 cases (20.2%). Although less frequent, Sanders type IV fractures consistently represented a clinically relevant subgroup and were associated with higher rates of post-operative complications and secondary surgical procedures.

Information regarding fracture exposure was available in 27 studies, encompassing 4185 fractures. Of these, 312 were open fractures, corresponding to 7.5% of reported cases. Studies directly comparing open and closed fractures consistently demonstrated higher infection rates in open fractures, ranging from 18% to 42%, compared with 2% to 14% in closed injuries.

Patient-related risk factors were inconsistently reported. Smoking status was available in 19 studies involving 2146 patients, with a mean prevalence of 34.6%. Diabetes mellitus was reported in 17 studies including 1982 patients, with a prevalence of 11.2%, while obesity (BMI > 30 kg/m^2^) was reported in nine studies with a prevalence of 18.5%. In all studies that evaluated these variables, smoking and diabetes mellitus were associated with an increased risk of post-operative infection (Table 3).

### 3.2. Surgical Management and Perioperative Variables

Surgical technique was reported in all included studies, covering 4638 fractures. Open reduction and internal fixation with plates and screws was the most frequently employed strategy, accounting for 3221 fractures (69.4%). Minimally invasive techniques were increasingly reported in more recent studies, including the sinus tarsi approach in 782 fractures (16.9%), percutaneous screw fixation in 381 fractures (8.2%), minimally invasive plate osteosynthesis in 173 fractures (3.7%), and intramedullary nail fixation in 81 fractures (1.8%).

The extended lateral L-shaped approach was described in 26 studies, predominantly in series published before 2015, and was associated with a higher incidence of wound-related complications.

Operative time was reported in 29 studies, with a weighted mean of 92.4 min and a range of 40 to 155 min. Time from injury to surgery was available in 24 studies, with a mean delay of 9.6 days (range, 2–22 days), largely influenced by soft tissue conditions.

Perioperative antibiotic prophylaxis was described in 28 studies. A single intravenous dose or prophylaxis lasting less than 24 h was administered in 75% of studies, whereas extended prophylaxis beyond 48 h was reported in 25% (Table 4).

### 3.3. Infection Rates and Microbiological Findings

Post-operative infection data were available in 38 studies, encompassing 4402 fractures. A total of 412 infections were reported, corresponding to an overall infection rate of 9.4%. Superficial surgical site infections accounted for 279 cases (6.3%), while deep infections were reported in 133 cases (3.0%).

Deep infections were strongly associated with adverse outcomes, including implant removal in 62% of cases, prolonged intravenous antibiotic therapy in all cases, and additional surgical procedures in 71%.

Microbiological data were available in 21 studies, comprising 198 isolates. *Staphylococcus aureus* was the most frequently isolated pathogen (46.5%), including methicillin-resistant strains in 14.1% of cases. *Escherichia coli* and *Pseudomonas aeruginosa* accounted for 18.2% and 16.7% of isolates, respectively, while other Gram-Negative organisms represented 18.6% (Table 5).

### 3.4. Management of Infectious Complications

Management strategies for infectious complications were reported in 35 studies. Superficial infections were generally managed with local wound care and short courses of oral or intravenous antibiotics. In contrast, all deep infections required surgical debridement and prolonged intravenous antibiotic therapy. Implant removal was necessary in 61.7% of deep infections, and negative pressure wound therapy was used in 36.8% of cases.

Orthoplastic procedures, including local or free flap coverage, were required in 41 cases, representing 30.8% of deep infections, particularly following extended lateral approaches (Table 6).

### 3.5. Complications and Further Surgical Procedures

Non-infectious complications were reported in 33 studies and included wound dehiscence in 6.8% of cases, flap necrosis in 4.1%, implant irritation or failure in 9.3%, and post-traumatic subtalar arthritis in 18.7%.

Secondary surgical procedures were frequently required, including implant removal in 512 cases (11.0%) and subtalar arthrodesis in 286 cases (6.2%). These procedures were significantly more common in patients with deep infections and Sanders type IV fractures. Functional outcomes, assessed using AOFAS or FFI scores in 22 studies, were consistently worse in patients who developed infections, with mean AOFAS differences ranging from 12 to 18 points, although statistical significance was not uniformly demonstrated (Table 7).

### 3.6. Risk Factors for Infection

Across the included studies, several factors were consistently associated with an increased risk of post-operative infection, including smoking, diabetes mellitus, open fractures, severe soft tissue injury, use of the extended lateral approach, and higher Sanders classification. Reported odds ratios ranged from 1.8 to 3.2 for smoking and from 2.1 to 4.5 for diabetes mellitus. Due to heterogeneity in study design and outcome reporting, a pooled quantitative analysis was not feasible.

## 4. Discussion

The present systematic review provides a comprehensive synthesis of the available evidence on infectious complications following surgical treatment of calcaneal fractures. By analyzing 40 studies published between 2001 and 2025, this review confirms that post-operative infection remains a clinically relevant complication, with an overall infection rate of approximately 9.4%. This incidence is consistent with previously reported series and recent focused analyses on calcaneal fracture surgery and wound complications [27,65]. A central finding of this review is the strong relationship between fracture severity and post-operative morbidity. Displaced intra-articular fractures represented the vast majority of cases, with Sanders type II and III fractures accounting for nearly 80% of classified injuries. However, Sanders type IV fractures, although less frequent, were consistently associated with higher rates of deep infection, secondary surgical procedures, and inferior functional outcomes. Severe comminution, impaired local vascularity, and prolonged operative times likely contribute to the higher susceptibility to infection observed in these cases.

Open fractures, while relatively uncommon in the included studies, demonstrated markedly higher infection rates compared with closed injuries. Infection rates in open calcaneal fractures ranged from 18% to 42%, consistent with previous trauma literature and recent systematic observations [30,66]. These data reinforce the importance of staged surgical protocols, meticulous soft tissue management, and appropriate timing of definitive fixation in high-energy calcaneal injuries.

Patient-related risk factors were inconsistently reported across studies; nevertheless, smoking and diabetes mellitus emerged as the most frequently analyzed comorbidities and were consistently associated with an increased risk of infection. Recent evidence has emphasized the role of systemic host factors, particularly metabolic disorders and impaired microcirculation, in predisposing patients to post-operative infectious complications [67]. The relatively high prevalence of smoking and diabetes observed in the present review further supports the need for careful preoperative risk stratification and patient counseling.

With respect to surgical management, open reduction and internal fixation with plates and screws remained the most commonly employed technique, particularly in older series. The extended lateral approach was widely used in earlier studies and has repeatedly been associated with higher rates of wound complications and infection. More recent literature, including focused analyses on minimally invasive strategies, has demonstrated a progressive shift toward less invasive approaches such as the sinus tarsi approach and percutaneous fixation [68]. Although direct comparisons were limited in the present review, this trend likely reflects an effort to reduce soft tissue trauma while preserving acceptable fracture reduction.

The overall infection rate observed in this review is comparable to those reported in recent focused studies on infection after calcaneal fracture surgery [69]. Importantly, while superficial surgical site infections were more frequent and generally manageable with conservative treatment, deep infections carried disproportionately severe consequences. In this review, deep infections required implant removal in over 60% of cases and additional surgical procedures in more than 70%, confirming the findings of recent dedicated analyses on deep infection following foot and ankle trauma [69].

Microbiological findings further underline the complexity of these infections. *Staphylococcus aureus*, including methicillin-resistant strains, was the most commonly isolated pathogen, followed by Gram-Negative organisms such as *Escherichia coli* and *Pseudomonas aeruginosa*. Similar pathogen distributions have been reported in recent studies focusing on fracture-related infection in foot and ankle surgery, supporting the need for broad-spectrum empiric antibiotic coverage until culture-specific therapy can be initiated [70].

Management strategies varied according to infection severity. Superficial infections were typically managed with local wound care and short courses of antibiotics, whereas deep infections universally required surgical debridement and prolonged intravenous antibiotic therapy. The frequent need for implant removal and negative pressure wound therapy reflects the anatomical constraints of the calcaneal region and the limited soft tissue envelope. Notably, nearly one-third of deep infections required orthoplastic procedures, particularly following extended lateral approaches, a finding also highlighted in recent multidisciplinary management studies [71].

Beyond infection, non-infectious complications such as wound dehiscence, flap necrosis, implant-related problems, and post-traumatic subtalar arthritis were commonly reported. Secondary procedures, including implant removal and subtalar arthrodesis, were particularly frequent in patients with deep infection and Sanders type IV fractures. Functional outcomes, assessed using AOFAS or FFI scores, were consistently worse in patients who developed infections, with clinically meaningful reductions reported across multiple studies, in line with previously published outcome analyses [72].

The findings of this review emphasize that infection prevention should remain a central goal in calcaneal fracture surgery. Optimization of modifiable patient-related risk factors, careful timing of surgery, and judicious selection of surgical approach appear crucial in reducing complications. In severe fracture patterns and high-risk patients, minimally invasive or staged strategies may offer advantages in terms of soft tissue preservation. Moreover, early recognition and aggressive management of deep infection are essential to limit long-term functional impairment.

### Study Limitations

This systematic review has several limitations. Most included studies were retrospective observational cohorts, inherently subject to selection bias and incomplete reporting. Considerable heterogeneity in study design, infection definitions, surgical techniques, and follow-up duration precluded quantitative meta-analysis. Patient-related risk factors and microbiological data were inconsistently reported, limiting the ability to perform pooled risk estimates. Functional outcomes were assessed using different scoring systems and were not uniformly reported. Finally, although risk of bias was assessed using the MINORS tool, variability in methodological quality across studies must be considered when interpreting the results.

## 5. Conclusions

Infectious complications remain a clinically relevant and challenging issue following surgical treatment of calcaneal fractures. This systematic review demonstrates that post-operative infections occur in approximately one out of ten surgically treated cases and are strongly influenced by fracture severity, soft tissue conditions, surgical approach, and patient-related comorbidities. Although superficial infections are more common and generally manageable with conservative measures, deep infections are associated with substantial morbidity, frequently requiring implant removal, prolonged antibiotic therapy, and additional surgical procedures, with a significant negative impact on functional outcomes.Severe fracture patterns, particularly Sanders type IV fractures, open injuries, and patients with risk factors such as smoking and diabetes mellitus, represent high-risk subgroups in whom careful preoperative assessment and tailored surgical strategies are essential. The progressive adoption of minimally invasive techniques reflects an ongoing effort to reduce soft tissue complications, although high-quality comparative data remain limited.Future research should focus on well-designed prospective studies with standardized definitions of infection and outcome measures, as well as on the identification of optimal surgical and perioperative strategies aimed at minimizing infectious complications and improving long-term patient outcomes after calcaneal fracture surgery.

## Figures and Tables

**Table 1:** Inclusion and Exclusion Criteria.

Inclusion Criteria	Exclusion Criteria
**Adult patients (≥18 years)**	Case reports
**Surgically treated calcaneal fractures**	Expert opinions
**Post-operative infection defined according to CDC SSI criteria**	Letters to the editor
**Fracture-related infections consistent with FRI definition (when reported)**	Previous systematic reviews or meta-analyses
**Minimum follow-up of 12 months**	Non-surgical treatment of calcaneal fractures
**Original clinical studies (RCTs, prospective or retrospective cohort studies)**	Incomplete or non-quantitative infection data
**Full-text available**	Lack of clear infection definition

**Table 2:** Study Characteristics, Patient Demographics and MINORS Score. (NR = not reported).

First Author	Year	Country	Study Design	Patients (n)	Fractures (n)	MINORS Score
**Soni et al. [26]**	2014	UK	Retrospective study	69	69	12/16
**Wallace et al. [27]**	2022	Texas	Retrospective study	105	100	12/16
**Basile et al. [28]**	2023	Italy	Prospective comparative study	54	54	20/24
**Rak et al. [29]**	2009	Czech Republic	Retrospective study	76	67	12/16
**Wang et al. [30]**	2018	China	Retrospective study	NR	681	12/16
**Lu et al. [31]**	2021	China	Retrospective study	900	900	12/16
**Lin et al. [32]**	2018	China	Retrospective study	129	129	12/16
**Patil et al. [33]**	2013	India	Retrospective study	238	226	12/16
**Makki et al. [34]**	2010	UK	Retrospective study	47	45	12/16
**Tennent et al. [35]**	2001	UK	Retrospective study	51	47	12/16
**Howard et al. [36]**	2003	Canada	Prospective comparative study	NR	161	20/24
**Koski et al. [37]**	2005	Finland	Retrospective study	148	126	12/16
**Ho et al. [38]**	2013	Taiwan	Retrospective study	53	50	12/16
**Ding et al. [39]**	2013	China	Retrospective study	490	479	12/16
**Court-Brown et al. [40]**	2019	UK/Netherlands	Retrospective study	178	178	12/16
**Cianni et al. [41]**	2022	Italy	Retrospective study	33	28	12/16
**Biz et al. [42]**	2016	Italy	Retrospective study	87	82	12/16
**W. Jianchuan et al. [43]**	2020	China	Retrospective study	35	35	12/16
**Jain S et al. [44]**	2013	China	Retrospective study	26 (22 unilateral + 2 bilateral)	24	12/16
**G. Vicenti et al. [45]**	2018	Italy	Retrospective study	42	42	12/16
**Ma et al. [46]**	2016	China	RCT	64	64	12/16
**Welck et al. [47]**	2015	UK	Prospective observational	33	30	14/16
**Driessen et al. [48]**	2022	Netherlands	retrospective study	81	72	12/16
**Moussa et al. [49]**	2022	French	retrospective study	24	24	12/16
**Backes et al. [50]**	2013	Netherlands	retrospective study	191	191	12/16
**Li et al. [51]**	2016	China	retrospective study	176	162	12/16
**Christin Schindler et al. [52]**	2021	Switzerland	Retrospective monocentric cohort study	129	114	12/16
**Nathaniel Rawicki et al. [53]**	2015	USA	Retrospective monocentric cohort study	17	17	12/16
**Wang et al. [54]**	2015	China	Retrospective monocentric cohort study	118	107	12/16
**Fitschen-Oestern et al. [55]**	2010	Germany	Prospective cohort study	37	37	14/16
**Piotr Sypien et al. [56]**	2025	Poland	Retrospective monocentric cohort study	48	48	12/16
**Zhao et al. [57]**	2019	China	Retrospective monocentric cohort study	142	142	12/16
**Dingemans et al. [58]**	2015	Netherlands	Retrospective monocentric cohort study	94	94	12/16
**Shams et al. [59]**	2019	Germany	Prospective cohort study	40	38	14/16
**A. K. Singh et al. [60]**	2013	India	Retrospective comparative study	390	390	12/16
**Takuya Usami et al. [61]**	2023	Japan	Retrospective comparative study, single-center	47	47	12/16
**Vladimír Popelka et al. [62]**	2019	Slovakia	Retrospective	162	145	12/16
**Hammam Kayali et al. [63]**	2024	Qatar	Retrospective comparative study, single-center	49	49	12/16
**P. Zeman et al. [64]**	2008	Czech Republic	Retrospective monocentric cohort study	61	49	12/16

**Table 3:** Patient Demographics and Fracture Characteristics.

Variable	Value
**Mean age (years)**	31.6–57
**Male sex**	75–90%
**Sanders classification reported**	31/40 studies (3912 fractures)
**Sanders type II**	1604 (41.0%)
**Sanders type III**	1517 (38.8%)
**Sanders type IV**	791 (20.2%)
**Open fractures**	312/4185 (7.5%)
**Smoking prevalence**	34.6%
**Diabetes mellitus prevalence**	11.2%
**Obesity (BMI > 30 kg/m^2^)**	18.5%

**Table 4:** Surgical Techniques and Perioperative Management.

Parameter	Findings
**Total surgically treated fractures**	**4638**
**ORIF (plates and screws)**	**3221 (69.4%)**
**Sinus tarsi approach**	**782 (16.9%)**
**Percutaneous screw fixation**	**381 (8.2%)**
**MIPO**	**173 (3.7%)**
**Intramedullary nail fixation**	**81 (1.8%)**
**Extended lateral approach**	**26 studies**
**Mean operative time**	**92.4 min (range 40–155)**
**Mean delay to surgery**	**9.6 days (range 2–22)**
**Antibiotic prophylaxis < 24 h**	**75% of studies**
**Antibiotic prophylaxis > 48 h**	**25% of studies**

**Table 5:** Infection Characteristics and Microbiology.

Variable	Findings
**Studies reporting infections**	**38**
**Total fractures analyzed**	**4402**
**Overall infection rate**	**412 cases (9.4%)**
**Superficial SSI**	**279 (6.3%)**
**Deep infections**	**133 (3.0%)**
**Implant removal in deep infections**	**62%**
**Additional surgery required**	**71%**
***Staphylococcus aureus***	**46.5%**
**MRSA**	**14.1%**
***Escherichia coli***	**18.2%**
***Pseudomonas aeruginosa***	**16.7%**
**Other Gram-Negative bacteria**	**18.6%**

**Table 6:** Management of Infections.

Treatment Strategy	Frequency
**Local wound care and antibiotics (superficial SSI)**	**Majority of cases**
**Surgical debridement (deep infection)**	**100%**
**Prolonged IV antibiotics**	**100%**
**Hardware removal**	**61.7%**
**Negative pressure wound therapy**	**36.8%**
**Flap reconstruction**	**30.8% of deep infections**

**Table 7:** Post-operative Complications and Outcomes.

Complication/Outcome	Incidence
**Wound dehiscence**	6.8%
**Flap necrosis**	4.1%
**Implant irritation or failure**	9.3%
**Post-traumatic subtalar arthritis**	18.7%
**Implant removal**	512 cases (11.0%)
**Subtalar arthrodesis**	286 cases (6.2%)
**Functional outcome (AOFAS/FFI)**	Worse in infected patients (−12 to −18 points)

## Data Availability

The original contributions presented in this study are included in the article/Supplementary Materials. Further inquiries can be directed to the corresponding author.

## Supplementary Materials

The following supporting information can be downloaded at: https://www.mdpi.com/article/10.3390/life16030528/s1, PRISMA Checklist.

## Abbreviations

AOFAS	American Orthopaedic Foot & Ankle Society
ARIF	Arthroscopically assisted reduction and internal fixation
BMI	Body mass index
CDC	Centers for Disease Control and Prevention
FFI	Foot Function Index
FRI	Fracture-related infection
MIPO	Minimally invasive plate osteosynthesis
MINORS	Methodological Index for Non-Randomized Studies
MRSA	Methicillin-resistant *Staphylococcus aureus*
NPWT	Negative pressure wound therapy
ORIF	Open reduction and internal fixation
PRISMA	Preferred Reporting Items for Systematic Reviews and Meta-Analyses
SSI	Surgical site infection
